# Comparing results of X-ray diffraction, µ-Raman spectroscopy and neutron diffraction when identifying chemical phases in seized nuclear material, during a comparative nuclear forensics exercise

**DOI:** 10.1007/s10967-017-5666-3

**Published:** 2018-01-24

**Authors:** Stina Holmgren Rondahl, Fabien Pointurier, Linnea Ahlinder, Henrik Ramebäck, Olivier Marie, Brice Ravat, François Delaunay, Emma Young, Ned Blagojevic, James R. Hester, Gordon Thorogood, Aubrey N. Nelwamondo, Tshepo P. Ntsoane, Sarah K. Roberts, Kiel S. Holliday

**Affiliations:** 1 CBRN Defence and Security, Swedish Defence Research Agency (FOI), Umeå, Sweden; 2 DAM, DIF, French Alternative Energies and Atomic Energy Commission (CEA), 91297 Arpajon, France; 3 Department of Chemistry and Chemical Engineering, Nuclear Chemistry, Chalmers University of Technology, Göteborg, Sweden; 4 French Alternative Energies and Atomic Energy Commission (CEA), CEA-Centre de Valduc, 21120 Is-Sur-Tille, France; 5 Australian Nuclear Science and Technology Organisation (ANSTO), New Illawarra Road, Lucas Heights, NSW 2234 Australia; 6 South Africa Nuclear Energy Corporation (NECSA) Pelindaba, 582, Pretoria, 0001 Gauteng South Africa; 7 Lawrence Livermore National Laboratory (LLNL), Livermore, CA 94551 USA

**Keywords:** XRD, µ-Raman Spectroscopy, Neutron diffraction, Phase identification, Nuclear forensics, Uranium oxide

## Abstract

This work presents the results for identification of chemical phases obtained by several laboratories as a part of an international nuclear forensic round-robin exercise. In this work powder X-ray diffraction (p-XRD) is regarded as the reference technique. Neutron diffraction produced a superior high-angle diffraction pattern relative to p-XRD. Requiring only small amounts of sample, µ-Raman spectroscopy was used for the first time in this context as a potentially complementary technique to p-XRD. The chemical phases were identified as pure UO_2_ in two materials, and as a mixture of UO_2_, U_3_O_8_ and an intermediate species U_3_O_7_ in the third material.

**Electronic supplementary material:**

The online version of this article (10.1007/s10967-017-5666-3) contains supplementary material, which is available to authorized users.

## Introduction

The fourth collaborative material exercise (CMX-4) organized by the Nuclear Forensics International Technical Working Group (ITWG) comprised a scenario where two samples had been confiscated after an alleged “simple possession” of a radioactive nature. A black powder (ES-1), approximately 3 g of sample, was found on a suspect at an international airport, and an item suspected to be a nuclear fuel pellet (ES-2) was subsequently found in a shed at the housing of the suspected person. Two years prior to these seizures another fuel pellet (ES-3) was seized by authorities at an abandoned warehouse in another country. More details about this exercise can be found in Ref. [1]. This includes the description of several other techniques for identification of physical and chemical characteristics of the seized materials, like isotopic composition, elemental composition, and date of the last separation. The results from all of these techniques were used to draw conclusions regarding similarities between, and the possible origin of, the three samples.

Reports were to be submitted to the exercise coordinators after 24 h, 1 week and 2 months after receipt of the samples. These timelines are in accordance with the IAEA Nuclear security recommendations [2].

Identifying the phases of the seized materials aids in pinpointing the origin of the materials (e.g., type of nuclear facility used for the production or handling of seized materials). A few laboratories that participated in the exercise used µ-Raman spectroscopy (µ-RS) and/or powder X-ray diffraction (p-XRD) for phase identification. One laboratory used neutron diffraction (ND) to identify the chemical phases in the powder sample (ES-1).

The techniques, µ-RS, p-XRD, and ND are all quick and easy to implement since they require a minimum of sample preparation. Moreover, µ-RS is very sensitive to slight changes on molecular environment and crystalline phase, as it is possible to simultaneously measure Raman active phonon modes in crystalline materials and Raman active vibrational modes in molecules. It is thus possible to both get a unique spectral fingerprint of different polymorphs of crystalline materials and spectral information from molecules in the measurement spot. Besides, all three techniques are practically nondestructive, even for microscopic objects. In the case of µ-RS care must be taken not to induce laser damage. Also, µ-RS has the specific advantage of being applicable to very small sample amounts (µm-sized particles), and in case of heterogeneous samples can be used to analyze micrometric details of the materials (e.g., some parts of the sample which differ in color or aspect compared to the main part of the material). In the past µ-RS was successfully applied to identify the main uranium compounds encountered in the nuclear industry [3–14]. Powder XRD is known to be an efficient tool for the phase analysis of nuclear compounds, although higher amounts of material are necessary as compared to µ-RS. A typical p-XRD pattern consists of a set of diffraction peaks of intensity *I* (in counts) located at reflective angles 2θ (in degrees) corresponding to lattice plane spacing, or reciprocal lattice vector *d*_*h*,*k*,*l*_, of crystallographic indices (*h*,*k*,*l*) as given by Bragg’s law:

1 $$n\lambda = 2d_{h,k,l} \sin (\theta )$$

where λ is the wavelength of the X-ray source (in Å) and *n* is a positive integer. This allows for identification of phase and relative composition (structural characterization) by matching the measured peaks at, in particular, 2θ in terms of the peak position and intensity with the patterns from the International Centre for Diffraction Data (ICDD), Crystallography Open Database (COD) or similar libraries [15–17]. To evaluate all sample diffraction patterns collected by p-XRD for the four laboratories the raw data [i.e., collected relative intensities at different angles (2θ)] was converted from 2θ to *d*_h,k,l_ according to Eq. (1). Powder XRD was earlier used to ascertain the phases measured by µ-RS when bibliographic information was limited [9, 13, 18, 19]. ND provides complementary information to p-XRD because neutrons interact with the point-like nucleus of an atom, whereas X-rays interact with the extended electron cloud surrounding the atom. The neutron interaction is not proportional to Z, so low Z atoms (such as oxygen) contribute significantly to the diffracted intensity in ND, whereas the diffracted intensity in p-XRD is dominated by the higher Z atoms (such as uranium). Furthermore, the point scattering of the neutron results in a much slower drop-off in intensity at high angles.

The aim of this paper is to show how µ-RS can be used to complement XRD, or in the absence of XRD when differentiating between the three materials used during CMX-4. In this paper results obtained by µ-RS will be presented and compared to the result obtained by p-XRD and ND, as the latter two are more established methods with a greater reference library. The instruments and methods used for phase identification of the three materials, and the results obtained with these techniques, will be presented, compared, and discussed. Specific concerns like homogeneity of the samples at the micrometer-scale and possible oxidization of the samples by the RS laser will be addressed.

## Instruments and methods

### Instruments

Main characteristics of the instruments; p-XRD used by laboratories (code-named) Vermeer, Pollock, Rembrandt, Cezanne, and Monet; µ-RS used by Vermeer and Pollock; and Echidna high-resolution powder neutron diffractometer (ND) used by Rembrandt [20] are presented in Tables 1, 2, 3.

**Table 1:** Characteristics of the five XRD instruments used in this study

Laboratory	Vermeer	Pollock	Rembrandt	Cezanne	Monet
Manufacturer and model	Bruker D2 phaser	Bruker D8 advance	Bruker D8 advance	Bruker D8 advance	Bruker D8 advance
Source and wavelength^a^	Cu X ray tube *k*_α1_: *λ* = 1.54060 Å	Mo X ray tube *k*_α1_: *λ* = 0.7093 Å *k*_α2_: *λ* = 0.71359 Å	Cu X ray tube *k*_α1_: *λ* = 1.540564 Å *k*_α2_: *λ* = 1.544390 Å	Cu X ray tube *k*_α1_: *λ* = 1.54060 Å	Cu X ray tube *k*_α1_ = 1.540598 Å
Device for reduction of the *k*_β_-peaks	Ni-foil	Zr-foil	N/A^b^	Ni-foil	Ni-foil
Goniometer radius θ/θ (mm)	282.2	250	173	217.5	300
Detector	1-dimensional Lynx Eye, PSD detector Angular aperture: 5° (fixed)	1-dimensional Vantec, PSD detector Angular aperture: 6°	LynxEye XE^b^ Angular aperture: 3.0°	1-dimensional Lynx Eye, PSD detector Angular aperture: 2.7°	1-dimensional Lynx Eye, PSD detector Angular aperture: 2.7°
Geometry	Bragg–brentano θ/θ	Bragg–brentano θ/θ	Bragg–brentano θ/θ	Bragg–brentano θ/θ	Bragg–brentano θ/θ
Primary slits	0.2 mm	0.2 mm	1 mm, 1.2°, 1 mm	0.2 mm	0.1 mm

^a^Emission profile validated by measurements on a certified reference material produced by Bruker, the corundum sample, or NIST SRM 1976 [21]

^b^Energy discriminating detector, no need for secondary monochromator or metal filters

**Table 2:** Characteristics of the neutron diffractometer used in this study

Laboratory	Rembrandt
Manufacturer and model	Echidna high-resolution powder diffractometer
Wavelength	1.622 Å
*d*-spacing range	0.8–14 Å
Sample tube	Vanadium cylinder 6 mm diameter 0.1 mm thick
Detector and operating temperature (°C)	^3^He gas-filled tubes at room temperature

**Table 3:** Characteristics of the two µ-Raman spectrometers used in this study

Laboratory	Vermeer	Pollock
Manufacturer and model	Horiba–Jobin–Yvon HR 800 UV	Renishaw ‘Invia’
Laser wavelength (nm)	514	514
	785	785
Laser characteristics (lasing medium)	Argon ion (514 nm)	Argon ion (514 nm)
	Diode semi-conductor (785 nm)	Diode semi-conductor (785 nm)
Spot size of laser	With ×100 objective: ~ 0.4 µm^2^	With ×100 objective: ~ 0.4 µm^2^
Gratings (lines/mm)	300 for 785 nm	1800 for 514 nm
	600 for 785 nm	1200 for 785 nm
	1200 for 785 nm	
	600 for 514 nm	
	1800 for 514 nm	
Spectral range (cm^−1^)	> 4000 (for 1800 lines/mm)	> 4000 (514 nm)
	Up to ~ 3500 (for 600 lines/mm)	Up to ~ 3200 (785 nm)
	Up to ~ 1700 (for 300 lines/mm)	
Focal distance of the spectrometer (cm)	80	25
Numerical aperture (NA)	0.25 for ×10	0.75 for ×50
	0.45 for ×50 long work distance	0.85 for ×100
	0.75 for ×50	
	0.9 for ×60 water immersion	
	0.9 for ×100	
	1.25 for ×100 oil immersion	
Output power (mW)	300 (785 nm)	300 (785 nm)
	50 (514 nm)	50 (514 nm)
Slit (µm)	N/A^a^	Motorized, from 20 to 65 µm
Detector and operating temperature (°C)	Peltier (air) cooled CCD( − 70 °C)	Peltier (air) cooled CCD(− 70 °C)
Typical integration time (range)	10 ms to infinity	10 ms to infinity
Objectives	×10, ×50, ×50 long work distance, ×60 water immersion, ×100, ×100 oil immersion	×5, ×20, ×50, ×100

^a^No slit, since the instrument is a true confocal microscope and a confocal hole is used to control the sampling volume

It should be mentioned that the RS used by Pollock and Vermeer are µ-RS, for which the laser beam is focused though an optical microscope. Consequently, very small areas (~1 µm^2^) are analyzed. At the Vermeer laboratory, the µ-RS equipped with a true confocal aperture allows spatially resolved measurements over a couple of µm along the lateral (depth) axis.

### Sample preparations and analytical procedures

The sample preparation and analytical conditions applied for XRD analyses are summarized in Table 4.

**Table 4:** Sample treatment and analytical data handling for XRD analyses

Laboratory	Vermeer	Pollock	Rembrandt	Cezanne	Monet
Sample holder	Bruker (PMMA) holders, rotated during analysis	Anton Paar – TTK 450 chamber, not rotated during analysis	Bruker Airtight^a^ holder (PMMA) with dome-type X-ray transparent cap, rotated during analysis	Bruker Airtight^a^ holder (PMMA) with dome-type X-ray transparent cap, not rotated during analysis	Bruker (PMMA) holders. Pellets rotated during analysis, powders not rotated during analysis
CMX-4 sample pre-treatment	A subsample of ES-1 powder loaded into shallow plastic holder. ES-2 & ES-3 were measured as pellets.	ES-1 powder loaded between two sealed Kapton sheets ES-2 & ES-3 were measured as pellets.	Samples were mounted in airtight specimen holders with a plastic dome cover. ES-1 was analyzed as received. ES-2 and ES-3 were analyzed as resin-mounted sub-samples of the two pellets.	ES-1 powder loaded into holder as received. ES-2 & ES-3 pellets first ground to powder to homogenous sample	ES-1, ES-2 and ES-3; analysed as received. Subsamples from pellets were ground into powders
Evaluation package	Proprietary EVA Software and PDF-2 reference database 2015 (ICDD)	Proprietary EVA Software and PDF-4 + reference database (ICDD)	X’Pert HighScore search/match data analysis software and PDF-2 reference database	Proprietary EVA Software and PDF-2 reference database 2007 (ICDD)	Proprietary EVA and TOPAS Software and PDF-2 database 2009 (ICDD)
*d* spacing analysis range	4.4–1.2	4.2–0.85	17–0.80	5.9–0.89	17–1.3 for solid pellets and 8.8–1.4 for powder samples
Acquisition time (min)	42 (2500 steps, 0.024° step size, 1 steps/s)	900 (2700 steps, 0.015°step size, 0.05 steps/s)	480 (2900 steps, 0.05^o^ step size, 0.1 steps/s)	460 (11,040 steps, 0.001^o^ step size, 0.4 steps/s)	Solids: 126 (3648 steps, 0.01, 918° step size, 0.5 steps/s) Powders: 109 (3128 steps, 0.01918° step, 0.5 steps/s)
XRD pattern refinement	Bruker EVA for semi-quantitative phase analysis, RIR method	Bruker EVA for semi-quantitative phase analysis, RIR method	GSAS-II^b^ freeware	Bruker EVA for semi-quantitative phase analysis, RIR method	Bruker EVA for semi-quantitative phase analysis, Bruker TOPAS^c^ for quantitative phase analysis

^a^The airtight sample holder is used by this laboratory to avoid risking contamination of the instrument and/or accidental inhaling of the radioactive material

^b^General structure analysis system-II crystal structure refinement

^c^Total pattern analysis solutions-software

Sample preparation and analytical conditions applied for µ-RS analyses are summarized in Table 5. For Pollock, it should be noted that only very low amounts of uranium can be handled in the laboratory and inside the instrument, dedicated to trace analysis of nuclear materials. Therefore, only small fragments (typically tens of µm), although regarded as macroscopic pieces, of the two original pellets were sampled and analyzed at Pollock. Pollock also analyzed micrometric particles directly sampled onto the pellets, before breaking them in several parts. The goal of these complementary analyses was to check for other possible chemical compositions than the one determined for the pellets (i.e., another uranium compound handled in the original nuclear facility that had been produced by a nuclear activity other than the one which led to pellet manufacturing). A special preparation procedure was used for these samples: sampling with cotton wipes swiped onto surfaces of the pellets, deposition onto graphite disk using a vacuum impactor, which aspires particles and deposits them onto a glassy carbon disk. Eventually, uranium particles were located at the disk’s surface by SEM and relocated inside the µ-RS using a mathematical calculation.

**Table 5:** Sample preparation techniques and analytical conditions for RS analyses

Laboratory	Vermeer	Pollock macroscopic fragments	Pollock surface micrometric particles
Sub-sampling and preparation	ES-3: one fragment (~0.5 g) after broken up into 4 pieces ES-2: entire pellet ES-1: transfer of ~0.01 g to a substrate using a 1 ml pipette tip	ES-2 and ES-3: several fragments (~10–100 µm) after breaking pellets sampled with sticky carbon tape ES-1: small tip in contact with the powder, then with a sticky carbon tape	ES-2 and ES-3: gently wiping surfaces of the pellets with cotton clothes. Extraction from cotton, deposition onto graphite disk, SEM localization
Substrate	CaF_2_ substrate for ES-1, ES-2 and ES-3 were measured directly on a glass plate	Sticky carbon tapes	Graphite disk
Laser used for the analysis (nm)	514	514	514
Power (mW)	Six for all samples 13 for time study of ES-3^b^	~2.5 (5%)^b^	~0.05 (0.1%)^b^, ~0.5 (1%)^b^ or ~2.5 (5%)^b^ depending on the particle size
Acquisition time (s)	60 30×60 at the same spot for ES-2 to evaluate possible oxidation caused by the laser irradiation	60 (6×10)	60 (6×10)
Number of measurements	20 each sample	20 each sample	20 particles for ES-2 20 particles for ES-3
Spectral range (cm^−1^)	200–1800^a^	100–1400^a^	100–1400^a^
Objective	×10 for ES-2 and ES-3 ×50 for ES-1	×100	×100
Background correction (Yes/No), method	Yes, background correction according to Zhang et al. [22]	Yes, cubic spline interpolation provided with Wire 3.4. software package	Yes, cubic spline interpolation
Curve fitting (Yes/No), algorithm	Yes, provided with LabSpec 6 software	Yes, provided with Wire 3.4 software package	Yes, provided with Wire 3.4 software package

^a^Peaks detected below 200 cm^−1^ are probably due to lattice vibrations or to light diffusion through the notch filter. They are not taken into account in data treatment

^b^Incident powers of the RS are adjusted thanks to attenuation filters, which allow transmission of a given percentage of the maximal power

Pollock and Vermeer both carried out uncertainty calculations on the positions of the Raman bands. Uncertainties are the quadratic combination of a systematic uncertainty of 0.5 cm^−1^ (estimated from repetitive measurements of the main band of silicon at 520.5 cm^−1^) and of the standard deviation calculated over all measurements (20 per sample). If not stated otherwise, all uncertainties are expanded uncertainties with a coverage factor *k* = 2, corresponding to an approximate 95 percent confidence interval.

No sample pretreatment was required for ND analysis of the powder. Approximately 1.7 g of the powder, as received, was loaded into a cylindrical vanadium can (6 mm diameter and 0.1 mm wall thickness), where it was fully illuminated by a 50 mm (V) × 20 mm (W) neutron beam. No sample rotation was required, as the combination of moderate beam divergence, high sample transparency, and relatively large quantity of sample ensured that a statistically large number of powder domains were in the diffracting condition at any given position.

## Results and discussion

### Results of the phase analysis of the pellet samples

#### p-XRD results for the two pellets

**Fig. 1:**
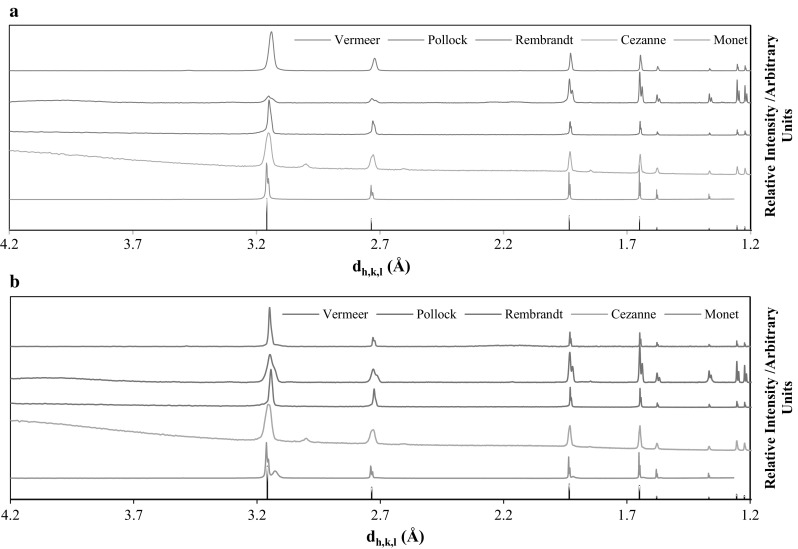
From top to bottom, spectra for ES-2 (**a**) and ES-3 (**b**) obtained by XRD analysis of macroscopic samples by Vermeer, Pollock, Rembrandt, Cezanne, and Monet. The reference spectrum for UO_2_ has been added (black bars) at the bottom of the graph

XRD pattern obtained for the two pellet samples (ES-2 and ES-3) by the five laboratories are given in Fig. 1. The diffraction patterns for these samples are very similar when comparing the results from all laboratories. Also, the diffraction peaks are very thin, which implies long-range ordering of the two materials. All laboratories observed a good match with the expected peak positions for UO_2_ centered-face cubic crystal phase (card PDF number 03-065-0285 [23]), indicating that both ES-2 and ES-3 are made of pure UO_2_, with a lattice parameter of 5.4710 Å.

No significant difference could be established between the ES-2 and ES-3 phases based on their p-XRD pattern, except for the diffraction pattern for ES-3 from Monet. A non-stoichiometric UO_2 + *x*_ (*x* = 0.25) was identified with its main peak at d-spacing 3.12 Å. The additional phase in ES-3 is thought to be due to aging on the surface of the pellet, which would result in an oxidized phase. When analyzing a crushed and powdered sub-sample of ES-3 there was no non-stoichiometric UO_2_ phase to be found.

All diffraction patterns, except the ones from Cezanne, observe double peaks throughout. These double peaks are *k*_α,2_ peaks from the X-ray source. They can be removed by the evaluation software. However, at *d* spacing 1.8, 2.6, and 3.0 Å, small peaks are visible for Cezanne. As those peaks are thought to result from the sample preparation, their phase identification was not performed. These peaks are not observed for any of the other four laboratories.

Worth noting is that the intensity observed, in the diffraction pattern obtained for Pollock, differ from the other four laboratories due to their use of Mo X-ray source instead of Cu. However, this is not a problem seeing as the work presented focuses on identification rather than quantitative analysis, where peak intensity would have an impact.

#### RS results for ES-2 and ES-3 pellets

**Fig. 2:**
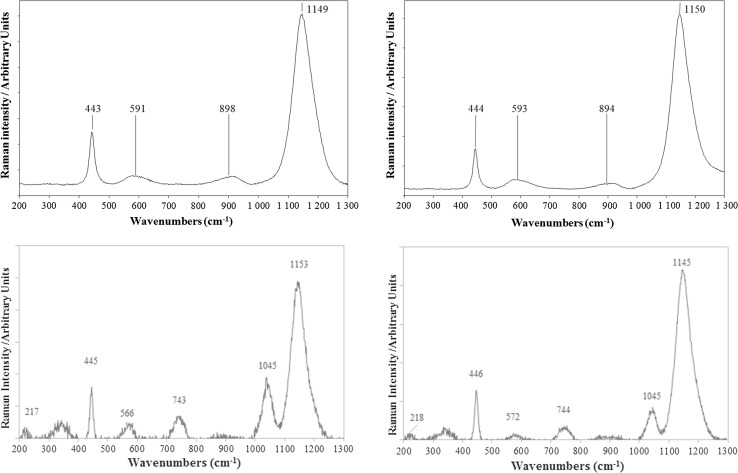
Average spectra obtained at Pollock by µ-RS analysis of 20 small fragments of the sample ES-2 (upper left) and ES-3 (upper right) and at Vermeer by µ-RS analysis of ES-2 (lower left) and ES-3 (lower right)

For both ES-2 and ES-3 pellet samples, Raman spectra obtained from measurements on different macroscopic fragments of various sizes (from ~10 to ~100 µm) (Pollock) or at different locations of the same fragment of the original pellets (Vermeer) are well-reproducible, the spectra can be found in Fig. S1 (supplementary information). Average spectra obtained by the two laboratories for both materials are given in Fig. 2. Wavenumbers of the bands detected and possible assignments are gathered in Table 6. A good agreement was obtained for bands univocally assigned to pure UO_2_ (445 and 1150 cm^−1^) by several authors [3–6, 8, 10, 11, 24–28] using lasers with wavelengths of 488, 514, 532 or 633 nm. However, spectra obtained by Vermeer show peaks typical of strongly oxidized UO_2_ (2.09 ≤ O/U ≤ 2.20) in both samples [6]. These bands at 222, 337, 744 cm^−1^ were not observed by Pollock. This phenomenon might be due to sample oxidation by the laser, in accordance with the findings of Allen et al. [3]. But, measurements performed by Vermeer at the same spot during 30 min (30×60 s, total delivered power of ~13 mW) for ES-3 show no significant change of the spectra along the experiment (see supplementary information Fig. S2). So these results suggest that the sample is not affected by the laser irradiation. As p-XRD analysis showed that the materials are pure UO2, another possible explanation lies in a surface oxidization phenomenon of the pellets for Vermeer. It might also be an artifact from the background subtraction due to high interferences from fluorescence. One way of avoiding such artifacts would be to perform analyses on raw spectra instead of background subtracted ones.

**Table 6:** Main Raman bands detected by Pollock and Vermeer for samples ES-2 and ES-3 in the 200–1300 cm^−1^ range. Uncertainties are expanded uncertainties (*k* = 2). Peaks that were not identified by the software but are visible after background correction have not been assigned an uncertainty. Wavenumber are expressed in cm^−1^. Bands mentioned in this table are detected for all of the 20 measurements carried out by each laboratory

Sample ID	Pollock: band wavenumber ± uncertainty	Vermeer: band wavenumber ± uncertainty	Possible assignment and reported range of wavenumbers
ES-2		217 ± 6	Not assigned but observed by some authors for U_3_O_8_^a^
		337	U_3_O_8_ A_1g_ O–U stretching bands^a^
	443 ± 2	445 ± 3	UO_2_ (U–O stretching T_2g_), range 445–450 cm^−1^
	591 ± 4	566 ± 8	UO_2_ (1LO phonons of the crystal), range 498–575 cm^−1^
		743 ± 7	U_3_O_8_ combination of two A_1g_ O–U stretching bands, range 751–763 cm^−1^
	898 ± 3	896	Not assigned but often observed for UO_2_
		1047 ± 6	
	1149 ± 2	1144 ± 7	UO_2_ (2LO phonons of the crystal), range 1149–1160 cm^−1^
ES-3		218 ± 7	Not assigned but observed by some authors for U_3_O_8_^a^
		337	U_3_O_8_ A_1g_ O–U stretching bands^a^
	445 ± 1	446 ± 3	UO_2_ (U–O stretching T_2g_), range 445–450 cm^−1^
	593 ± 5	572 ± 8	UO_2_ (1LO phonons of the crystal), range 575–498 cm^−1^
		744 ± 9	U_3_O_8_ combination of two A_1g_ O–U stretching bands, range 751–763 cm^−1^
	894 ± 3	896	Not assigned but often observed for UO_2_
		1045 ± 7	
	1150 ± 1	1153 ± 13	UO_2_ (2LO phonons of the crystal), range 1149–1160 cm^−1^

^a^According to Manara and Renker [6], Senanayake et al. [8]

However, there is no evident difference between samples ES-2 and ES-3 that can be observed using µ-RS.

#### Pollock—RS results for the µm-size particles sampled at the surfaces of the pellets

As mentioned above, particle analyses were carried out by Pollock by µ-RS on uranium particles sampled from the surfaces of the pellets by gently wiping the top of the pellets with a cotton cloth. Particles were then deposited on graphite disks. On each disk, 20 uranium-bearing particles were identified by SEM (“Quanta 3D”, FEI, Eindhoven, The Netherlands) with sizes ranging from 2 to 10 µm. Two categories of particles were evidenced by SEM imaging: (i) single “all-in-one-block” particles with typical size, (ii) agglomerates of sub-µm-size particles embedded in a non-definite matrix.

**Fig. 3:**
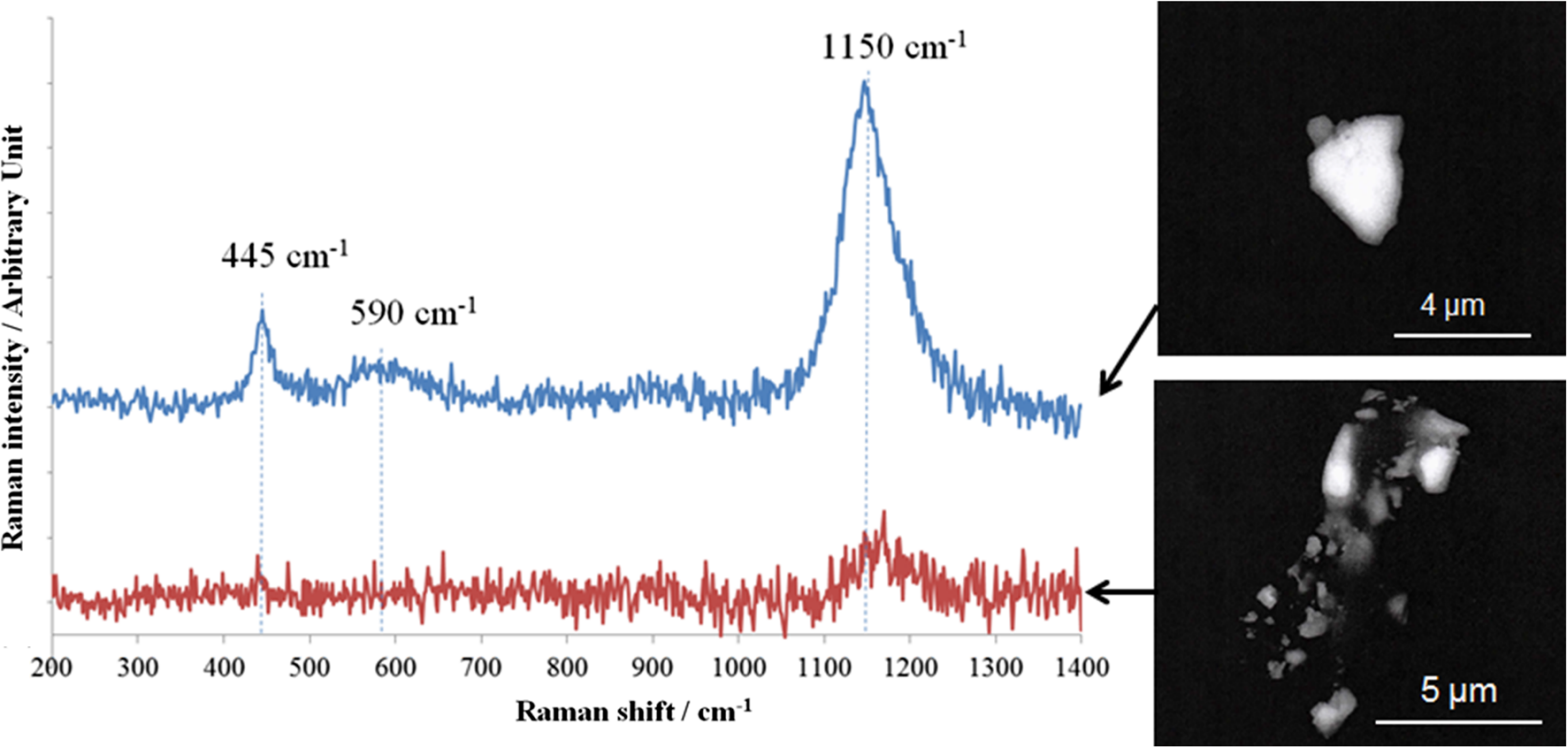
Typical examples of Raman spectra obtained at Pollock for an all-in-one-block particle (upper blue spectrum and associated SEM image) and for an agglomerate of sub-micrometric particles (lower red spectrum and associated SEM image). Both particles were sampled at the surface of the ES-3 pellet

All Raman analysis of the all-in-one-block particles of both samples ES-2 and ES-3 led to neat spectra, obviously characteristic of UO_2_ (see Fig. 3) and similar to the ones obtained from macroscopic fragments of the pellets. Analyses were much more difficult for agglomerates, due to difficulty in focusing the laser beam onto sub-µm objects, the low amounts of uranium contained into individual sub-particles, and a very high background, most likely due to fluorescence. No other explanation was found to explain such background. Its origin probably lies in the matter in which uranium particles were embedded. As a result, Raman analyses were unsuccessful for a few agglomerates. For the other agglomerates, only the band at ~1150 cm^−1^, which is the most intense one of the UO_2_ spectrum with the 514 nm-laser, was detected (Fig. 3).

The conclusion is that the chemical composition of the particles sampled at the surface of the two pellets ES-2 and ES-3 are similar to the bulk composition of the two pellets (i.e., UO_2_) evidenced by the same laboratory (Pollock) with the same µ-RS instrument and analytical conditions.

### Results for the powder sample

#### XRD results for the powder material

**Fig. 4:**
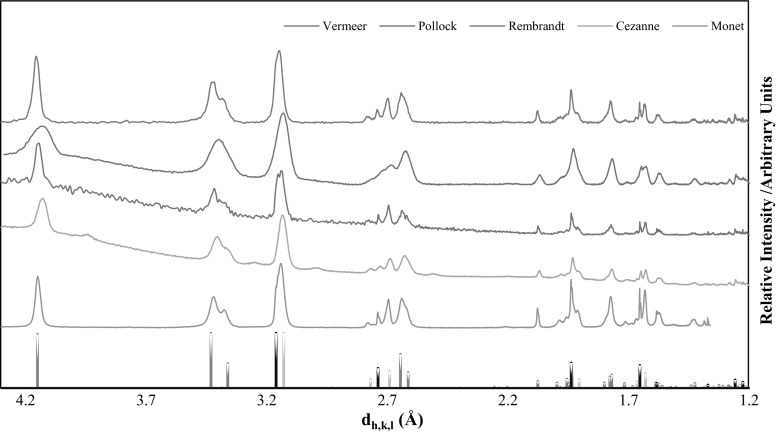
From top to bottom, spectra obtained by XRD analysis of the sample ES-1 by Vermeer, Pollock, Rembrandt, Cezanne and Monet. At the bottom of the figure reference diffraction pattern for U_3_O_8_ (dark gray), U_3_O_7_ (light gray) and UO_2_ (black) are provided

The XRD pattern obtained for the powder sample (ES-1) by the five laboratories are given in Fig. 4. The multi-phase diffraction pattern was highly complex. Some laboratories found it difficult to assign phases to the diffraction pattern due to its complexity and the presence of a large amorphous “hump” at low angle (high lattice plane spacing, *d*_*h*,*k*,*l*_). The amorphous signal was attributed to the use of a plastic dome sample holder. By comparing the *d*_*h*,*k*,*l*_ with positions referenced by the ICDD, the following compounds are detected: α-U_3_O_8_, centered-face orthorhombic crystal phase (PDF card number 00-031-1424, dark gray bars [29]); β-U_3_O_7_, quadratic crystal phase (PDF card number 00-042-1215, light gray bars [30]) and UO_2_, centered-face cubic crystal phase (PDF card number 03-065-0285, black bars [23]). With these data it is possible to say that the crystallographic structure of ES-1 differs from the one of ES-2 and ES-3. ES-1 has been identified as a mixture of different uranium oxides, U_3_O_8_, U_3_O_7_, and UO_2_. It is also possible that the peaks observed in the spectra might originate from another intermediate species of uranium oxide e.g., β-U_64_O_143_ (UO_2 + *x*_ where *x* = 0.23, PFD card 04-009-6397 [31]) or U_64_O_36_ (~ UO_1,75_, PDF card 04-006-7446 [32]) because the crystalline structures of these phases are quite similar it is difficult to draw any definitive conclusions regarding this intermediate species.

#### ND results for the powder material

**Fig. 5:**
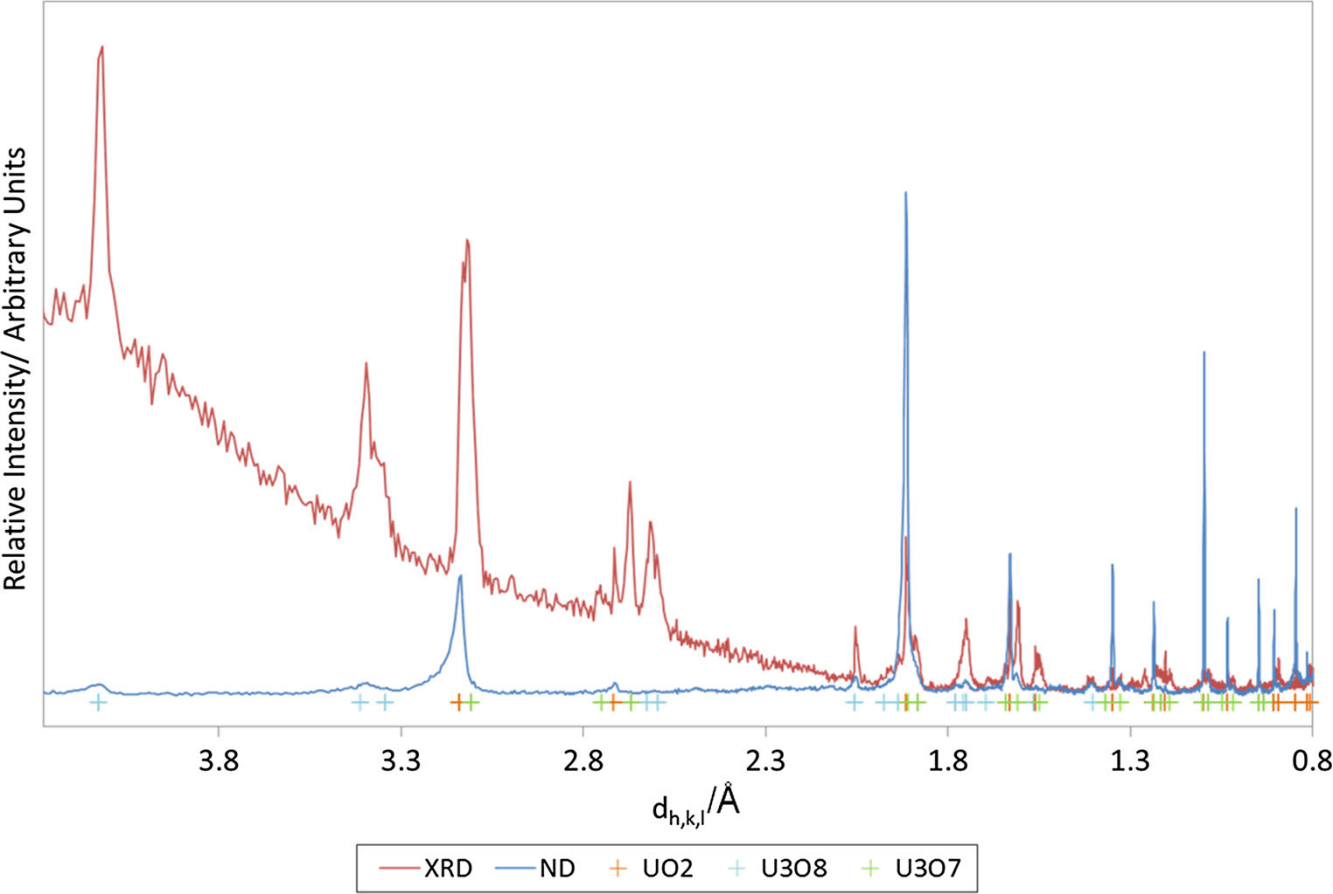
Comparison of ND and p-XRD patterns of ES-1 measured by Rembrandt. Orange crosses correspond to peaks of UO_2_ (PDF-03-065-0285 [24]), blue crosses correspond to peaks of U_3_O_8_ (PDF-01-074-2101 [33]) and green crosses correspond to peaks of U_3_O_7_ (PDF-00-042-1215 [27]). (Color figure online)

ND results for ES-1 are given in Fig. 5, compared with the p-XRD results obtained by Rembrandt. The combined p-XRD and ND patterns confirm the presence of the three phases UO_2_, U_3_O_8_ and U_3_O_7_ in ES-1.

#### µ-RS results for the powder material

According to observation performed with optical and electronic microscopes, ES-1 is composed of micrometer-sized and mm- sized particles. According to a visual observation by Pollock, with the optical microscope attached to the RS, sizes of the particles analyzed by RS were between ~1 and ~5 µm. The Pollock analysis, although a µ-RS with a thin spot size was employed, may measure more than one particle in each analyzed spot because sampled particles were very close to each other.

Detected Raman bands and their proposed assignments are listed in Table 7. The Raman spectra obtained by the two laboratories for all of the 20 analyses can be seen in the supplementary information (Fig. S3).

**Table 7:** Main Raman bands for the sample ES-1 in the 200–1300 cm^−1^ range detected by Pollock and Vermeer. Uncertainties are expanded uncertainties (*k* = 2). Peaks that were not identified by the software but are visible after background correction have not been assigned an uncertainty. Wavenumber are expressed in cm^−1^

Pollock: band wavenumber ± uncertainty (rate of detection)	Vermeer: band wavenumber ± uncertainty (rate of detection)	Possible assignment and reported range of wavenumbers
239 ± 4 (19/20)	230 ± 3 (17/20)	U_3_O_8_ (vibration not assigned), range 230–241 cm^−1^
330 ± 6 (19/20)	336 ± 17 (18/20)	U_3_O_8_ (U–O stretching A_1g_) range 336–351 cm^−1^,
372 ± 6 (15/20)	378	*Not assigned*
417 ± 4 (20/20)		U_3_O_8_ (U–O stretching A_1g_), range 405–412 cm^−1^
454 ± 5 (20/20)	451	UO_2_ (U–O stretching T_2g_), range 445–450 cm^−1^
499 ± 6 (20/20)		U_3_O_8_ (U–O stretching E_g_), range 474–493 cm^−1^
587 ± 2 (5/20)		UO_2_ (vibration not assigned), range 575–498 cm^−1^
646 ± 7 (20/20)	612	U_3_O_8_ (overtones of U–O stretching A_1g_ and E_g_), range 638–640 cm^−1^
742 ± 3 (16/20)	760 ± 10 (18/20)	U_3_O_8_ (U–O–U–O stretching), range 738–753 cm^−1^
804 ± 3 (17/20)		U_3_O_8_ (overtones of U–O stretching A_1g_ and E_g_), range 798–811 cm^−1^

Most of the detected Raman bands for ES1 are typical bands commonly assigned to U_3_O_8_, in the range 233–241 cm^−1^ [3, 8–11, 25], 336–351 cm^−1^ [3, 5, 8–11, 25], 405–412 cm^−1^ [3, 5, 8–11, 25], 638–640 cm^−1^ [3, 9], 738–753 cm^−1^ [3, 5, 8, 9, 11, 25], and 798–811 cm^−1^ [3, 5, 9–11, 25]. It should be noted that Raman bands at approximately 233–640 cm^−1^ and approximately 650–900 cm^−1^ are overlapping in most of the µ-Raman spectra obtained at Vermeer and it is therefore difficult to assign bands in this region.

Furthermore, the main band commonly assigned to UO_2_ (at ~ 450 cm^−1^) is also systematically detected by Pollock and it is visible as part of overlapping Raman bands for this region in µ-RS from Vermeer. It should be noted that the very intense peak observed at ~1150 cm^−1^ for the two UO_2_ pellets is no longer observed in the case of ES-1 as this band corresponds to a phonon vibration of pure and homogeneous well-crystallized UO_2_ material.

Another band detected at 499 ± 6 cm^−1^ by Pollock is close to a medium-intensity band, observed in the literature [3, 5, 8–11, 25] in the range 474–493 cm^−1^ for U_3_O_8_ (U–O stretching E_**g**_**),** and was then initially assigned to U_3_O_8_. Raman analysis of U_3_O_7_ is poorly documented in the literature. Allen et al. [3] provide a reference spectrum for β-U_3_O_7_ with a characteristic band at ~500 cm^−1^. Unfortunately, this spectrum has a poor resolution, so that this band is very close to the U_3_O_8_ bands in the 474–493 cm^−1^ region. So the shoulder detected at 499 ± 6 cm^−1^ by Pollock can be attributed either to U_3_O_8_ or to U_3_O_7_. More generally, Raman spectra of U_3_O_8_ and of the intermediate species U_3_O_7_ and U_4_O_9_ show too much likeness to be distinguished due to the low-resolution in the µ-RS spectra obtained at the µm scale.

**Fig. 6:**
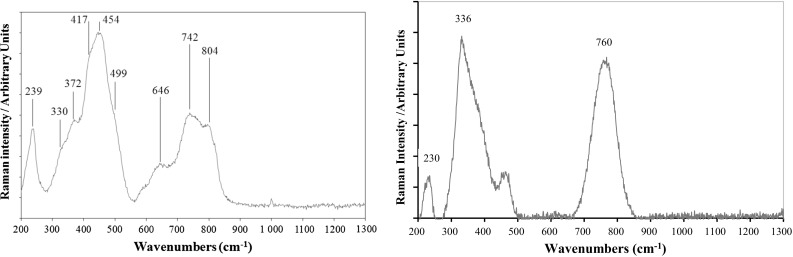
Average Raman spectra obtained by Pollock (left) and by Vermeer (right) for the sample ES-1

However, significant differences for ES-1 were observed between the spectra obtained by the two laboratories. This can be seen in the average spectra given in Fig. 6. More precisely, some bands detected by Pollock are not observed by Vermeer, like the bands at ~417, ~499, and ~804 cm^−1^. This is rather surprising as these bands are among the most frequent and most intense (especially a band at ~410 cm^−1^) detected for U_3_O_8_. However, both laboratories, independently drew the conclusion that ES-1 is made of a mixture of UO_2_ and U_3_O_8_, as enough bands assigned to the two species were detected for all analyzed particles.

Regarding reproducibility of the spectra, all spectra from Vermeer appear to be similar and are visually well-reproducible. On the contrary, Pollock spectra show significant visual differences even if most of the bands are detected in all of the spectra, especially the bands usually assigned to U_3_O_8_. Actually, relative intensities of the detected bands are highly variable from one analyzed spot to the other. It should be mentioned that, due to the small size and uneven surface of the analyzed objects, bands are very broad and determination of the band position is not achieved with a good reproducibility and accuracy. This lack of reproducibility might also be due to sample inhomogeneity at the particle’s level. The better reproducibility of the Vermeer spectra may be due to a larger spot size, which leads to the analyses of a higher amount of material, and thus, of more homogeneous micro-samples.

The literature suggests that the presence of U_3_O_8_ may result from a partial oxidization of UO_2_ after moderate heating under the laser beam [3, 8, 11]. However, detection of the same significant proportion of U_3_O_8_ with very low laser power invalidates this hypothesis.

### Discussion

#### Comparison between results obtained by µ-RS and p-XRD

Regarding the pellet samples (ES-2 and ES-3), µ-RS results obtained by Pollock are in very good agreement with results provided by p-XRD analysis. Results obtained by Vermeer are slightly biased towards an oxidized uranium oxide, probably due to difficulties in background subtraction or accidental surface oxidation.

Regarding the powder sample (ES-1), µ-RS results obtained by both Vermeer and Pollock are in good agreement with p-XRD results, as analyses with both techniques show that the ES-1 sample is made of a mixture of UO_2_ and U_3_O_8_. Powder XRD analysis by Pollock also revealed the possible presence of the β-U_3_O_7_ phase, which was not observed using µ-RS. It was not identified as β-U_3_O_7_ mainly because the Raman spectrum of β-U_3_O_7_ is not well-described in the literature. But when revisiting the results after the XRD analysis, the 499 ± 1 cm^−1^ band is significantly closer to the band for β-U_3_O_7_—500 cm^−1^ as reported by Allan et al. [3] —than that of U_3_O_8_. However, the Raman spectra obtained from particulate material have had a poor quality (low signal-to-noise ratio and broad bands) so it was difficult to draw any conclusions on the presence of another phase from this peak alone.

However, both techniques give complementary information. RS provides information essentially related to the surface of the sample, whereas p-XRD gives the chemical phase of the bulk material. Also, µ-RS requires a significantly lower amount of material than p-XRD; an analysis can be carried out on a µm-sized particle. Important to note is that there are XRD techniques available that are able to measure small amounts of sample, but these require a different kind of instrumentation. For example, it is possible to measure single particles using µ-XRD. But because µ-XRD requires a highly focused incident beam, which can be obtained at a synchrotron, for example, it is hardly standard instrumentation in any laboratory [34–36]. Surface and bulk information will normally be concordant if the sample is broken and the analysis by µ-RS is performed on enough (here 20 analyses for each sample) randomly chosen spots on a face representative of the inner material (which does not undergo surface oxidization). Also, the micrometric spatial resolution of µ-RS allows studying the homogeneity of the sample at a micrometer scale. Additionally, great care must be taken in sample preparation of the samples to avoid any chemical modification of the sample surface (i.e., oxidization or reduction by means of chemical reagents or thermal treatment). This means that µ-RS must be performed directly and as quickly as possible on the materials, or the samples must be stored in an environment that does not affect their chemistry.

#### Comparison of results obtained by ND and XRD measurements at Rembrandt

ND produced a superior high-angle diffraction pattern relative to p-XRD, which assisted in confirming oxidized phases. The high angle peaks of the ND pattern were more resolved and higher in intensity than those from p-XRD, making them more amenable to successful refinement, if the data were collected at the right conditions, to determine weight fractions of the different phases present.

The sealed vanadium sample holder used for ND was transparent to neutrons, and did not contribute to the pattern as well as satisfied the safety requirements.

ND is less likely to be easily accessible to nuclear forensics laboratories than p-XRD; however, the results obtained by Rembrandt show that if it is available, ND can be a complementary technique to p-XRD. ND could be most useful in situations where a superior higher-angle diffraction pattern is required, sample preparation requirements of p-XRD are likely to induce artefacts in the diffraction pattern, or the p-XRD pattern is unlikely to be of sufficient quality to be amenable to quantitative analysis.

#### Contribution to the determination of the origin of the materials

Findings of the p-XRD and µ-RS analyses suggest that, unlike samples ES-2 and ES-3, which exhibit the same UO_2_ phase, the ES-1 (powder) is an oxidized sample. The data suggested that an oxidation process (e.g., by heating) had been initiated, turning UO_2_–U_3_O_8_. Moreover, an incomplete oxidation process would explain the different phases identified by p-XRD and µ-RS.

## Conclusion and perspectives

This paper shows that µ-RS, XRD and ND techniques provided useful and coherent information on chemical phases present in three nuclear materials, two objects which looked like nuclear fuel pellets and one powder, in the framework of an international exercise on nuclear forensics. Results of all three techniques were in good agreement: similar phases were detected even if µ-RS is performed on significantly lower amounts of samples. This work demonstrated that µ-RS can be used as a highly effective screening tool in nuclear forensics. It reliably detects the various discreet phases present in uranium oxide samples. In a more general sense, µ-RS and XRD can be regarded as complementary techniques for in-depth nuclear forensic analyses. On the one hand, µ-RS is fast and easy to implement. It requires only minute amount of material; has the capability to identify chemical phases even in amorphous materials; ans allows the study of homogeneity at the µm-level, for µ-RS; and analysis of specific micrometric details. RS provides information related to the surface of the samples because of its limited depth penetration into uranium oxides. In contrast, XRD allows quantification of the various chemical phases present in the material and, thanks to the analysis of a larger amount of sample, provides representative information of the bulk composition of the studied material. µ-RS data can be used to complement or substitute for XRD analysis, as long as caution is used when drawing conclusions from the data seeing as µ-RS does not penetrate as deep into the sample. The complementary nature of XRD and ND can assist in positive identification of intermediate phases and potentially the accurate determination of weight fractions of phases present in nuclear forensic samples. In the near future, these techniques will certainly be used in forthcoming nuclear forensic exercises, carried out on other types of samples. So the laboratories will gain more experience and knowledge within their respective capabilities whether it be µ-RS, XRD or ND, for identification of chemical phases in seized nuclear materials.

## Electronic supplementary material

Below is the link to the electronic supplementary material.

Supplementary material 1 (DOCX 884 kb)
