# Supplementary material for: Comparing results of X-ray diffraction, µ-Raman spectroscopy and neutron diffraction when identifying chemical phases in seized nuclear material, during a comparative nuclear forensics exercise
Source: J Radioanal Nucl Chem. 2018 Jan 24;315(2):395–408. doi: 10.1007/s10967-017-5666-3 (PMC5820405; doi:10.1007/s10967-017-5666-3)
Supplement: Supplementary file 1 — Supplementary material 1 (DOCX 884 kb) [file 10967_2017_5666_MOESM1_ESM.docx]

# Supplementary material for - Comparing results of X-ray diffraction, µ-Raman spectroscopy and neutron diffraction when identifying chemical phases in seized nuclear material, during a comparative nuclear forensics exercise


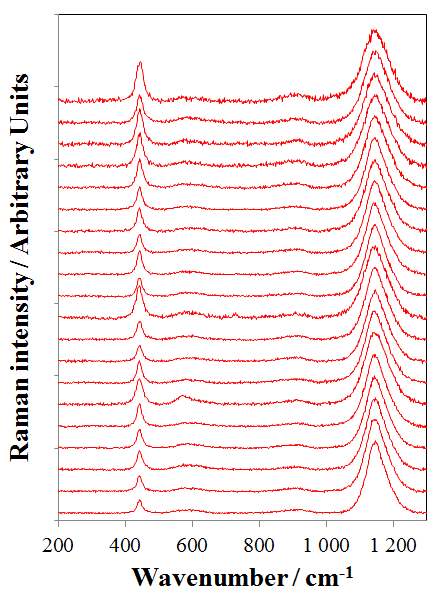


**Fig. S1** Raman spectra obtained at CEA for 20 fragments from pellet ES2.

**Fig. S2**  Raman spectra obtained by FOI for one single measurement spot on ES3 during 30 minutes (60 s per measurement). No visible distortion of the Raman bands is observed during the measurements, contrary to the findings of Allen et al (Allen 1987).

| 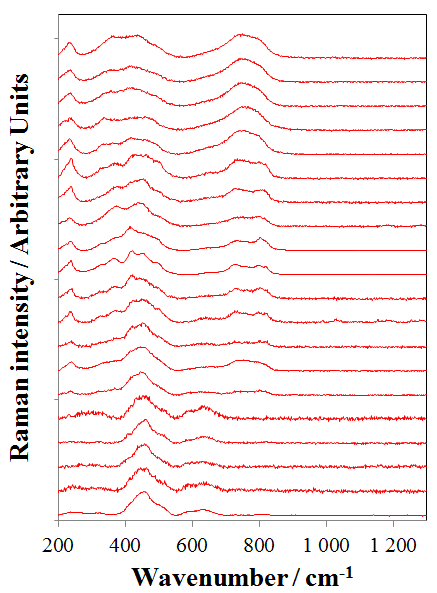 |  |
| --- | --- |

**Fig. S3** Raman spectra obtained at CEA (left) and FOI (right) for 20 analysis of the sample ES1 (powder).
